# Latest outcomes of transcatheter left atrial appendage closure devices and direct oral anticoagulant therapy in patients with atrial fibrillation over the past 5 years: a systematic review and meta-analysis

**DOI:** 10.1007/s12928-022-00839-1

**Published:** 2022-01-30

**Authors:** Keiichi Takeda, Yusuke Tsuboko, Kiyotaka Iwasaki

**Affiliations:** 1 Cooperative Major in Advanced Biomedical Sciences, Joint Graduate School of Tokyo Women’s Medical University and Waseda University, Waseda University, 2-2 Wakamatsucho, Shinjuku, Tokyo, 162-8480 Japan; 2 Waseda Research Institute for Science and Engineering, Waseda University, Tokyo, Japan; 3 Department of Modern Mechanical Engineering, School of Creative Science and Engineering, Waseda University, Tokyo, Japan; 4 Department of Integrative Bioscience and Biomedical Engineering, Graduate School of Advanced Science and Engineering, Waseda University, Tokyo, Japan; 5 Institute for Medical Regulatory Science, Comprehensive Research Organization, Waseda University, Tokyo, Japan

**Keywords:** Atrial fibrillation (AF), Direct oral anticoagulants (DOAC), Left atrial appendage closure (LAAC), Device-related thrombus (DRT), Meta-analysis

## Abstract

Left atrial appendage closure (LAAC) are emerging treatment for patients with atrial fibrillation (AF). However, data on the safety, efficacy, and medications for LAAC devices in patients with AF are lacking. We aimed to investigate the incidence of all-cause mortality, stroke, and major bleeding in AF patients with LAAC devices and DOACs. Moreover, we aimed to investigate the incidence rate of device-related thrombus (DRT) and the medications used in the management of AF patients with LAAC devices to gain insights into achieving better outcome. Based on a literature search using PubMed, EMBASE, Cochrane Library, and Web of Science databases between January 2015 and December 2020, eight LAAC device studies that used WATCHMAN and Amulet, and three DOAC studies that used rivaroxaban, with a total of 24,055 AF patients (LAAC devices, *n* = 2855; DOAC, *n* = 21,200), were included. A random-effects model was used to incorporate heterogeneity among studies. The pooled incidence of events per person-years were as follows: all-cause mortality, 0.06 (95% confidence interval [CI] 0.02–0.10) for WATCHMAN, 0.04 (95% CI 0.00–0.14) for Amulet, and 0.03 (95% CI 0.01–0.04) for rivaroxaban; stroke; 0.02 (95% CI 0.00–0.04) for WATCHMAN, 0 for Amulet, and 0.01 (95% CI 0.01–0.02) for rivaroxaban; major bleeding, 0.04 (95% CI 0.02–0.06) for WATCHMAN, 0.02 (95% CI 0.00–0.06) for Amulet, and 0.02 (95% CI 0.01–0.03) for rivaroxaban. The incidence rate of DRT was 2.3%, and complications were reported in 9%. The incidence of all-cause mortality, stroke, and major bleeding were similar between LAAC devices and DOACs. The rate of complications was acceptable, and those of DRT were lower than the average incidence reported in previous studies. However, further follow-up is needed. Concomitant anticoagulant and antiplatelet therapies should be further evaluated to find the optimal regimen for AF patients with LAAC devices.

## Introduction

The effectiveness of direct oral anticoagulants (DOACs) and left atrial appendage closure (LAAC) devices in preventing stroke in patients with AF has been recognized in recent years [1]. However, comparisons of LAAC devices and anticoagulants based on the latest clinical data are not properly understood when compared with studies on DOACs. Currently, the main LAAC devices used for patients with AF include WATCHMAN and Amulet. Both devices are approved in the US and the EU, however only WATCHMAN is approved in Japan [2].

As with interventional devices, such as those used in percutaneous coronary intervention and endovascular treatment of peripheral vessels, new LAAC devices require proper anticoagulant management and regular follow-ups [3, 4]. Information on device-related thrombus (DRT) is considered important. Some meta-analyses have reported the benefits and superiority of using LAAC devices by comparing randomized controlled trials (RCTs) of DOAC and LAAC devices [5, 6]. However, systematic reviews and meta-analysis of the latest LAAC devices and antithrombotic therapy have not been adequately conducted.

In this study, we conducted a comprehensive literature analysis through systematic reviews and meta-analyses of research data published over the last 5 years to indirectly compare the safety and efficacy of LAAC devices and DOACs. Moreover, we investigated the incidence of DRT in patients with AF who were treated with LAAC devices and current medication management strategies to gain insights into achieving better clinical outcome in AF patients with LAAC devices.

## Methods

### Search methods

This systematic review and meta-analysis were performed in accordance with the Preferred Reporting Items for Systematic Reviews and Meta-analysis (PRISMA) guidelines [7]. Studies regarding LAAC devices and DOACs published between January 2015 and December 2020 were investigated with a thorough search of the PubMed, EMBASE, Cochrane Library, and Web of Science databases. The first search was performed by two authors, using the terms “atrial fibrillation” AND (“left atrial appendage closure” OR “left atrial appendage occluder”) for LAAC, and “atrial fibrillation” AND (“Dabigatran” OR “Rivaroxaban” OR “Edoxaban” OR “Apixaban”) AND “real-world prospective study” for DOAC.

### Inclusion and exclusion criteria

**Fig. 1:**
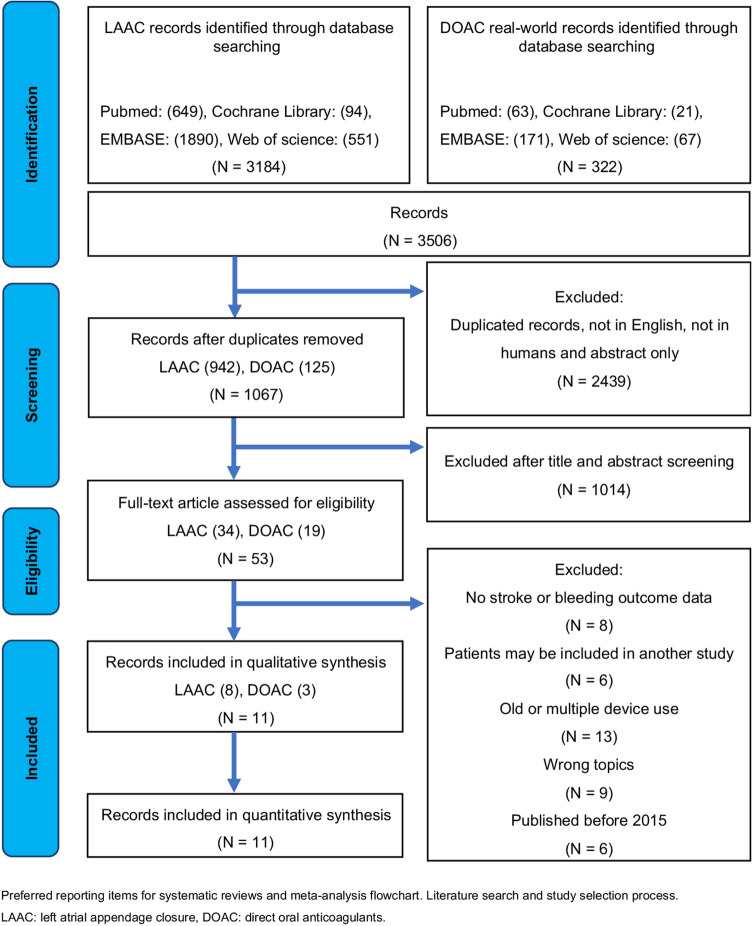
Flowchart of preferred reporting items for systematic reviews and meta-analysis. Literature search and study selection process. *LAAC* left atrial appendage closure, *DOAC* direct oral anticoagulants

Two independent authors determined whether the studies met the inclusion criteria and resolved discrepancies by a joint review and consensus. The study selection process is shown in Fig. 1.

The inclusion criteria were (a) publications in the English language, (b) sample size of > 40 patients with AF, (c) a minimum average follow-up period of 3 months, (d) inclusion of data on all-cause mortality, stroke, and major bleeding events, and (e) inclusion of complications related to the device or procedures. The exclusion criteria were (a) ongoing trials, (b) either LAAC devices or DOACs not being commercially available, (c) studies published before 2015, (d) simultaneous comparison of transcatheter aortic valve implantation, catheter ablation, and valve surgery, (e) inclusion of devices that used a non-femoral venous approach, including thoracoscopic or surgical approaches, (f) manuscript types, such as meta-analysis, network meta-analysis, systematic review, or case review, and (g) papers that were a sub-analysis of a previous study.

### Quality assessment

We evaluated the quality of all selected studies using the Standards for Reporting of Diagnostic Accuracy (STARD) guidelines [8] and calculated a score for quality. For quality assessment of each study, we used the Cochrane risk of bias tool [9]; two authors assessed the quality independently, and any disagreement was resolved after reaching a mutual consensus. The details of the studies and quality assessment of the STARD score are shown in Table 1. The Cochrane risk of bias summary is shown in Table 2.

**Table 1:** Selected studies for meta-analysis

Study	Study period	Country	Design	Device	Patients	Statistical analysis method	STARD score
LAAC studies							
Aonuma et al. [14]	2017, Feb–2017, Jul	Japan	M Pro SA	WATCHMAN	42	Intention-to-treat	27
Xue et al. [15]	2012, Feb–2017, Jan	Germany	S Retro Non-R	WATCHMAN	300	*t*-test Fisher’s exact test	22
Mazzone et al. [16]	2012, May–2015, Oct	Italy	M Pro Non-R	WATCHMAN	151	Kaplan–Meier	22
Boersma et al. [17]	2013, Oct–2015, May	Netherlands and 12 other countries	M Pro Non-R	WATCHMAN	1025	Kaplan–Meier	23
Saw et al. [18]	2013, May–2015, Oct	Canada	M P Non-R	WATCHMAN	106	Chi-square Fisher’s exact *t*-test	20
Sahiner et al. [19]	2015, Sep–2018, Mar	Turkey	S Retro Non-R	Amulet	60	Kolmogorov–Smirnov test Student’s *t*-test Mann–Whitney *U* test	23
Landmesser et al. [20]	2015, Jun–2016, Sep	Germany	M Pro Non-R	Amulet	1088	Kaplan–Meier	23
Masoud et al. [21]	2014, Oct–2016, Dec	United Kingdom	S Pro Non-R	Amulet	83	Kaplan–Meier	23
DOAC studies							
Ikeda et al. [22]	2012, Apr–2014, Jun	Japan	M Pro Non-R	Rivaroxaban	9578	Kaplan–Meier	28
Shimokawa et al. [23]	2012, Nov–2014, Jun	Japan	M Pro Non-R	Rivaroxaban	7141	Kaplan–Meier	26
Camm et al. [24]	2012, Jun–2013, Dec	Europe, Israel, and Canada	M Pro Non-R	Rivaroxaban	6478	Kaplan–Meier	28

*S* single-center, *M* multicenter, *Pro* prospective, *Retro* retrospective, *P* pooled, *SA* single-arm, *Non-R* non-randomized

**Table 2:** Cochrane risk of bias summary of the meta-analysis

Study	Bias				Applicability concerns		
	Patient selection	Index test	Reference standard	Flow and timing	Patient selection	Index test	Reference standard
Aonuma et al. [14]	L	L	L	L	L	L	L
Xue et al. [15]	L	L	L	U	L	L	L
Mazzone et al. [16]	L	L	H	U	L	L	L
Boersma et al. [17]	L	L	L	H	L	L	L
Saw et al. [18]	L	L	U	H	L	L	L
Sahiner et al. [19]	L	L	U	H	L	L	L
Landmesser et al. [20]	L	L	L	U	L	L	L
Masoud et al. [21]	L	L	L	L	L	L	L
Ikeda et al. [22]	L	L	L	U	L	L	L
Shimokawa et al. [23]	L	L	L	U	L	L	L
Camm et al. [24]	L	L	L	U	L	L	L

*L* low risk, *H* high risk, *U* unclear

### DRT

Based on the results of a previous DRT related study on LAAC devices, the incidence rate of DRT found by transesophageal echocardiography (TEE) testing at the 45 days, 6 months, or 12 months follow-up after implantation were within 3–7% [10]. Therefore, in this study, we have defined this as the acceptable limit.

### Statistical analysis

We conducted a standard meta-analysis using the proportions and corresponding standard errors in the inverse variance method [11]. The meta-analysis was conducted using a random-effects model to incorporate heterogeneity among studies. *I*-squared (*I*^2^) was used to quantify heterogeneity, and Tau-squared (*τ*^2^) was used as an estimate of the between-study variance in the random-effects meta-analysis, which was performed using the DerSimonian and Laird estimator for the *τ*^2^ and the Clopper–Pearson confidence intervals for each study. The meta-analysis was conducted for stroke, major bleeding, all-cause mortality, and DRT. We used forest and funnel plots to visualize the results of the meta-analysis and to investigate the existence of publication bias, respectively. Statistical analysis was conducted using the R language and environment for statistical computing [12] and the R package “meta” [13].

## Results

### Analyzed studies

The data from the past 5 years on WATCHMAN (WATCHMAN, Boston Scientific), Amplatzer Amulet (Amulet, Abbott Laboratories), and DOACs were compiled and analyzed in this study. We retrieved 3506 studies using the selected search words for LAAC devices (3184 studies) and DOACs (322 studies). Fifty-three studies (LAAC devices, 34 and DOACs, 19) were finally included after screening for duplicates, non-English papers, and abstract-only articles. Further, after checking for eligibility, eight studies on LAAC devices and three registry documents on DOACs were also included in this meta-analysis (Fig. 1).

In total, 2855 patients were included from all studies, and the range of the sample size among the selected studies was 42–1088. Among these, WATCHMAN was used for 1624 patients over five studies [14–18] and Amulet was used for 1231 patients over three studies [19–21]. In the DOAC group, a total of 21,200 patients from three studies (with sample sizes between 4838 and 9578 patients) that used rivaroxaban were included [22–24]. The overall male enrollment was 62% (LAAC device groups, 62%; DOAC group, 62%), except for the study by Sahiner et al. [19], (female = 58.3%). The average follow-up periods for the LAAC device groups and DOAC groups were 13 and 17 months, respectively (Table 3).

**Table 3:** Characteristics and outcomes of selected studies

	LAAC studies								DOAC studies		
Author	Aonuma et al. [14]	Xue et al. [15]	Mazzone et al. [16]	Boersma et al. [17]	Saw et al. [18]	Sahiner et al. [19]	Landmesser et al. [20]	Masoud et al. [21]	Ikeda et al. [22]	Shimokawa et al. [23]	Camm et al. [24]
Device/drug	W	W	W	W	W	A	A	A	R*	R*	R**
Patients	42	300	151	1025	106	60	1088	83	9578	4838	6784
Age	72.5 ± 8.8	75.1 ± 5.7	73 ± 8	73.4 ± 8.9	74.8 ± 7.7	72.3 ± 20.1	75 ± 9	76 ± 8.2	73.2 ± 9.8	71.6 ± 9.4	71.5 ± 10
Male	35	203	88	584	66	25	707	56	5921	3275	4016
Female	7	97	63	441	40	35	381	27	3657	1563	2768
Male sex (%)	83.3	67.7	58.2	56.9	62.2	41.6	64.9	67.4	61.8	67.6	59.1
CHADS_2_	2.5 ± 1.3	NA	2.3 ± 1.3	NA	2.8 ± 1.2	2.75 ± 2.25	NA	NA	2.2 ± 1.3	2.1 ± 1.3	2.0 ± 1.3
CHA_2_DS_2_-VAS_C_	3.6 ± 1.6	3.8 ± 1.5	3.7 ± 1.3	4.5 ± 1.6	4.3 ± 1.5	4.61 ± 2.61	4.2 ± 1.6	4 ± 1 (2–7)	3.4 ± 1.6	3.4 ± 1.7	3.4 ± 1.7
HAS-BLED	2.9 ± 1.1	3.5 ± 1.1	3.3 ± 1.1	2.3 ± 1.2	3.2 ± 1.2	4.32 ± 3.32	3.3 ± 1.1	NA	1.5 ± 1.1	1.4 ± 0.9	NA
Follow-up	6 months	637 ± 398	16 months	12 months	210 ± 182 days	21 ± 15 months	11.1 ± 2.6 months	12 months	300 ± 119 days	897.1 ± 206 days	1 year
AF type											
Paroxysmal	35.7%	32.3%***	42%		32.1%	21.6%		21.7%	33.6%	44.8%	40.6%
Persistence	21.4%	–	14%	–	–	78.3%	–	78.3%	35.8%	–	13.6%
Permanent	42.9%	67.7%	44%		67.9%	–		–	24.5%	–	27.0%
Other									6.1%	55.2%	18.7%
Stroke	1	11	5	NA	0	0	2	NA	128	171	43
	2.4%	3.7%	3.3%	NA	0.0%	0.00%	0.18%	NA	1.34%	3.53%	0.63%
All-cause mortality	0	35	5	91	4	NA	2	8	202	281	118
	0.0%	11.67%	3.3%	8.88%	3.8%	NA	0.18%	10.0%	2.11%	5.81%	1.74%
Major bleeding	1	19	6	26	5	1	4	7	143	215	128
	2.4%	6.33%	4.0%	2.54%	4.7%	1.67%	0.37%	8.8%	1.49%	4.44%	1.89%

*W* WATCHMAN, *A* Amulet, *R* rivaroxaban

*10/15 mg, **20 mg, ***Paroxysmal + persistence

### All-cause mortality

**Fig. 2:**
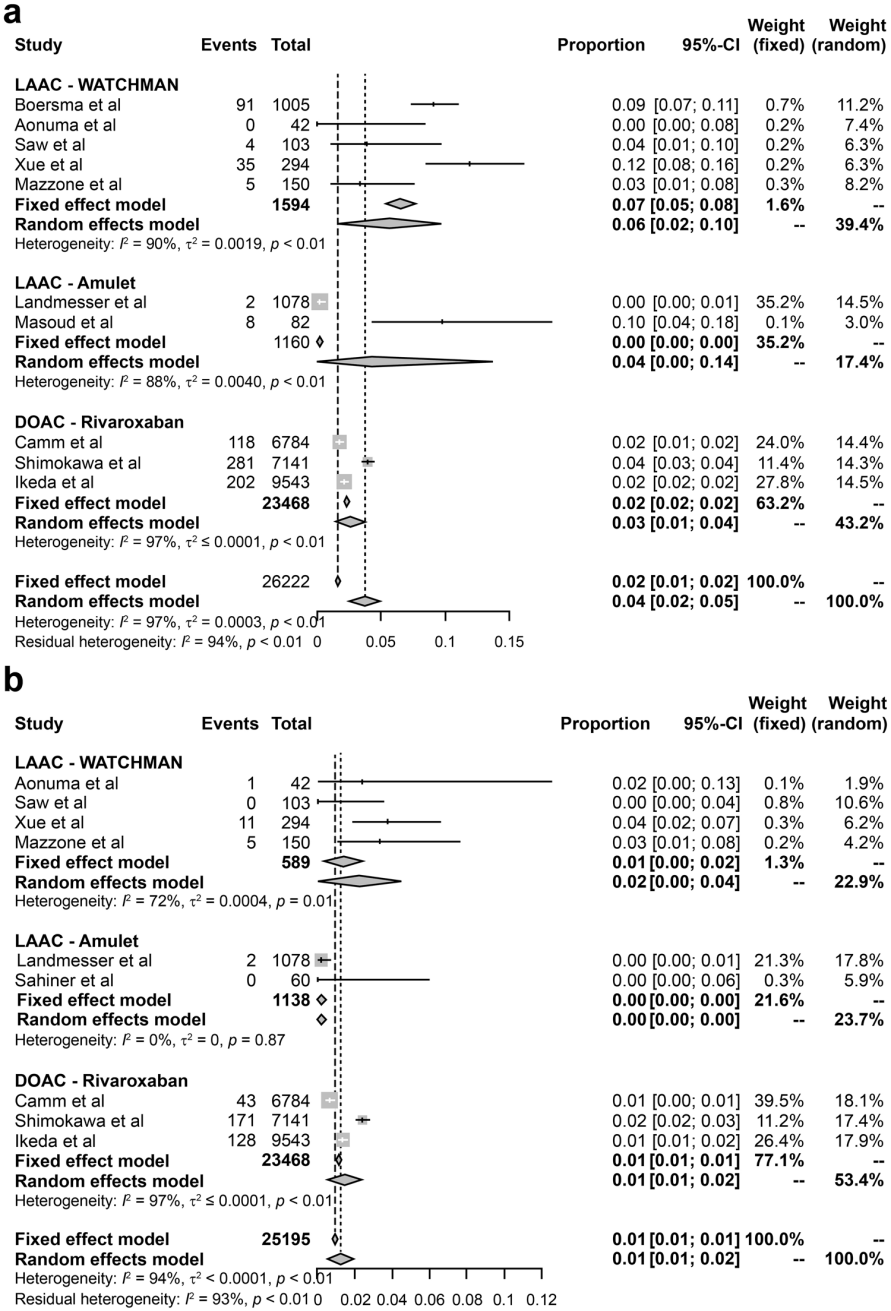

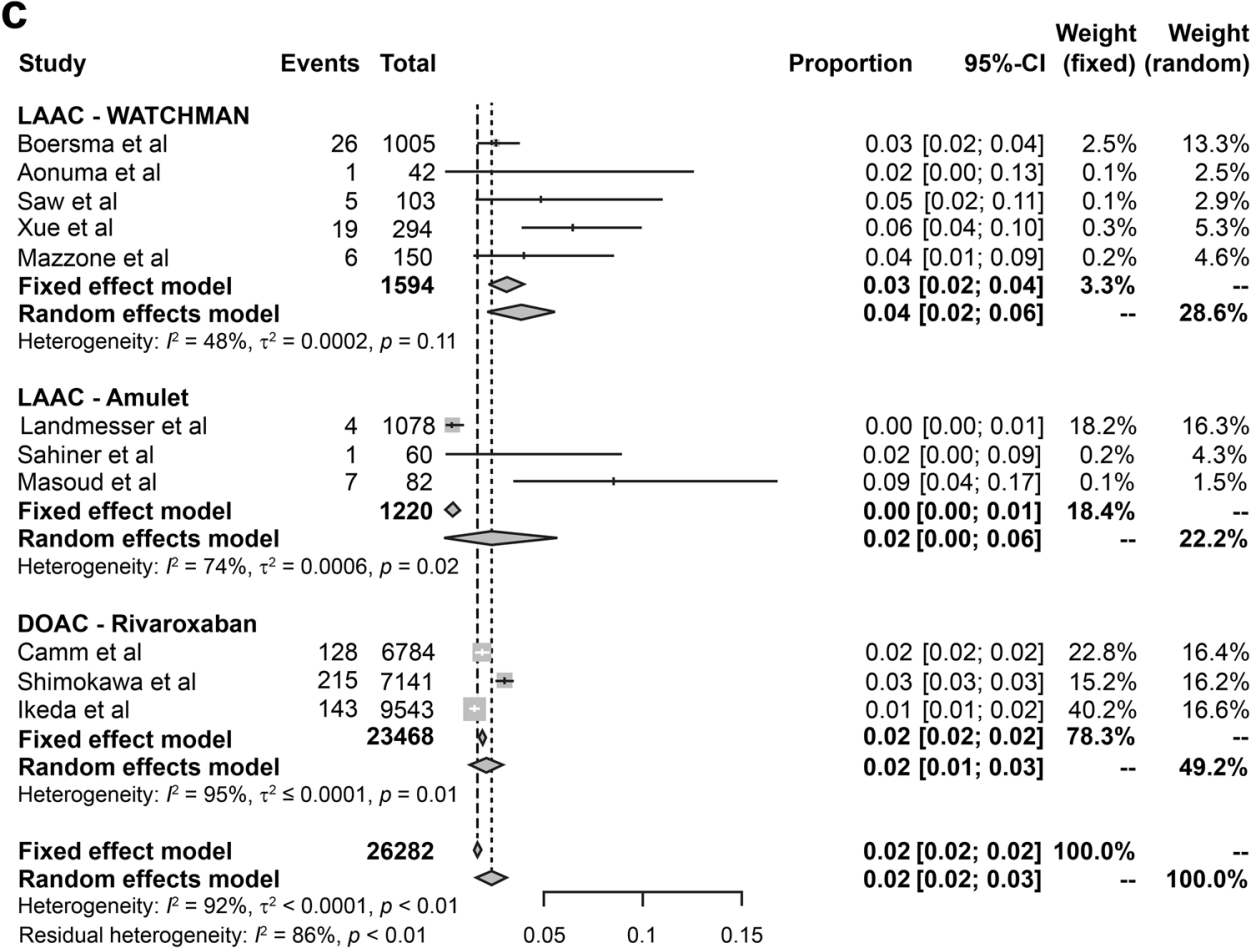
Forest plot of all-cause mortality, stroke, and major bleeding rates in studies on WATCHMAN, Amulet, and rivaroxaban. *CI* confidence interval, *DOAC* direct oral anticoagulants. **a** All-cause mortality rate, **b** stroke rate, **c** major bleeding rate

All-cause mortality was reported in seven LAAC device (WATCHMAN, 5; and Amulet, 2) and three DOAC studies, accounting for 2754 and 23,468 patients, respectively. The incidence of pooled all-cause mortality rates of WATCHMAN, Amulet, and rivaroxaban were 0.06 (95% confidence interval [CI] 0.02–0.10), 0.04 (95% CI 0.00–0.14), and 0.03 (95% CI 0.01–0.04) events per person-years, respectively (Fig. 2a). Significant heterogeneity was observed in all-cause mortality of studies on LAAC devices and DOACs (26,222 person-years; *I*^2^ = 97).

### Stroke prevention

Stroke prevention was reported in seven LAAC device (WATCHMAN, 5; Amulet, 2) and three DOAC studies, comprising 1727 and 23,468 patients, respectively. The incidence of pooled stroke rates of WATCHMAN, Amulet, and rivaroxaban were 0.02 (95% CI 0.00–0.04), 0, and 0.01 (95% CI 0.01–0.02) events per person-years, respectively (Fig. 2b). Significant heterogeneity was observed in stroke prevention of studies on LAAC devices and DOACs (25,195 person-years; *I*^2^ = 94).

### Major bleeding

Major bleeding was reported in eight LAAC device (WATCHMAN, 5; Amulet, 3) and three DOAC studies, with 2814 and 23,468 occurrences, respectively. The incidences of pooled major bleeding rates of WATCHMAN, Amulet, and rivaroxaban were 0.04 (95% CI 0.02–0.06), 0.02 (95% CI 0.00–0.06), and 0.02 (95% CI 0.01–0.03) events per person-years, respectively (Fig. 2c). Significant heterogeneity was observed regarding major bleeding in studies on LAAC devices and DOACs (26,282 person-years; *I*^2^ = 92).

**Fig. 3:**
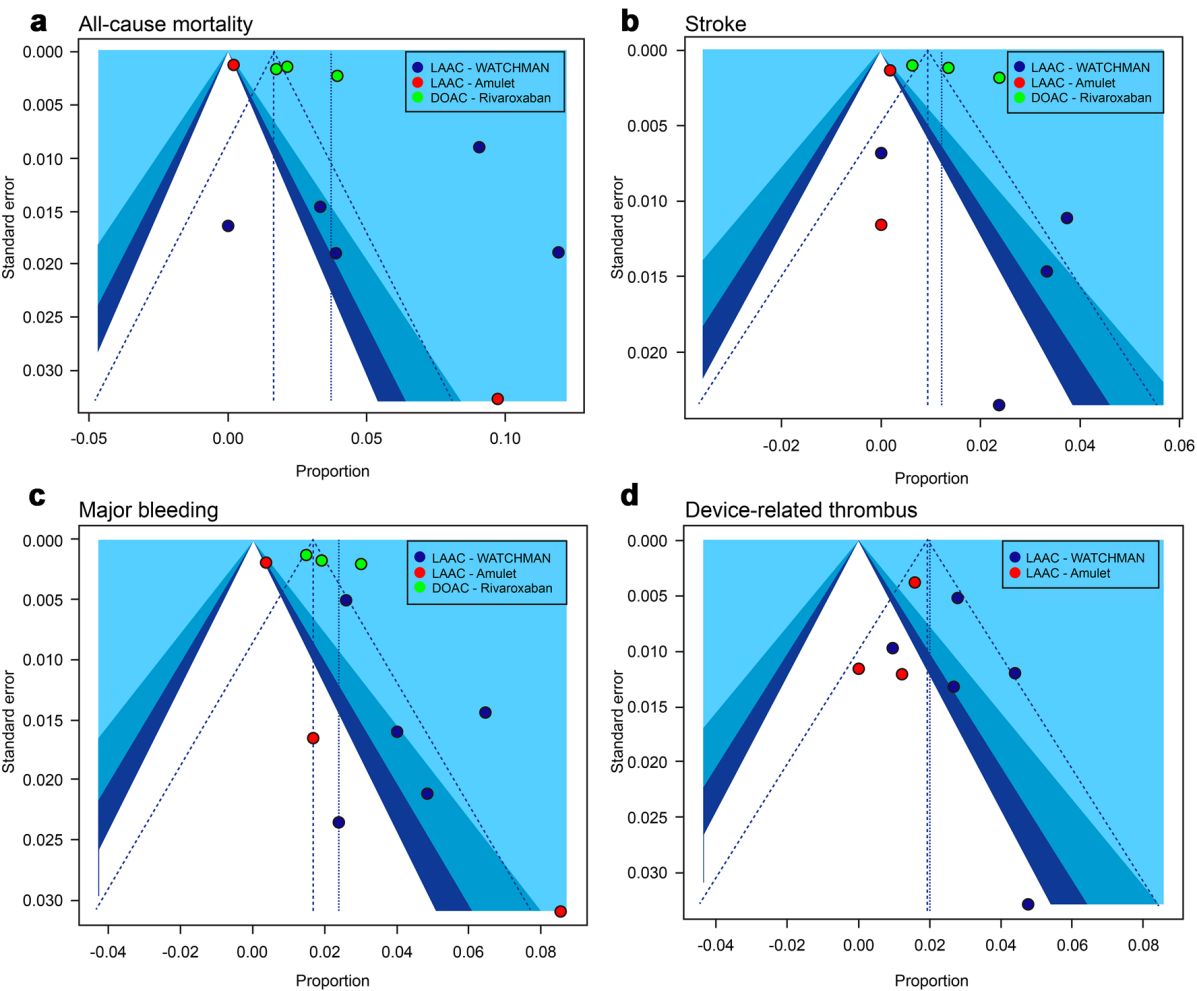
Funnel plots of all-cause mortality, stroke, major bleeding, and device-related thrombus events in studies on WATCHMAN, Amulet, and rivaroxaban. **A** All-cause mortality, **B** stroke, **C** major bleeding, **D** device-related thrombus. DOAC, direct oral anticoagulants

The funnel plot analysis indicated the presence of publication bias for all-cause mortality, stroke, and major bleeding, though not for DRT (Fig. 3).

### Periprocedural results

The procedural success rates of overall LAAC devices, WATCHMAN, and Amulet were 98.8% (2814/2855 cases), 98.5% (1594/1624), and 99.3% (1220/1231), respectively. Forty-one cases were unsuccessful because of structural anomalies. The rate of adverse events of LAACs were 2.8% (80/2814) and comprised the following: death, 0.4% (9/2370); stroke, 0.1% (3/2479); total pericardial effusion with cardiac tamponade, 1.0% (27/2732); major bleeding, 1.5% (37/2501); device embolization, 0.3% (7/2520); and device thrombosis, 0.2% (3/1414) (Table 4).

**Table 4:** Complications and adverse events during the procedural period within 7 days

	WATCHMAN					Amulet		
Author	Aonuma et al. [14]	Xue et al. [15]	Mazzone et al. [16]	Boersma et al. [17]	Saw et al. [18]	Sahiner et al. [19]	Landmesser et al. [20]	Masoud et al. [21]
Patients	42	300	151	1025	106	60	1088	83
Implanted	42	294	150	1005	103	60	1078	82
SR (%)	100.0%	98.0%	99.3%	98.0%	97.2%	100.0%	99.0%	98.8%
Failure	0/0.0%	6/2.0%	1/0.7%	20/2.0%	3/2.8%	0/0.0%	10/0.9%	1/1.2%
AE	0/0.0%	NA	5/3.3%	33/3.2%	2/1.9%	NA	35/3.2%	5/6.0%
Death	0/0.0%	NA	NA	4/0.4%	1/0.9%	1/1.7%	2/0.2%	1/1.2%
Stroke	0/0.0%	1/1.3%	NA	0/0.0%	NA	0/0.0%	2/0.2%	NA
TIA	0/0.0%	0/0.0%	NA	1/0.1%	NA	NA	2/0.2%	NA
PE/Tam	0/0.0%	4/1.3%	2/1.3%	3/0.3%	2/1.9%	3/5.0%	13/1.2%	NA
MB	0/0.0%	0/0.0%	NA	9/0.9%	NA	NA	26/2.4%	2/2.4%
FP	0/0.0%	NA	NA	NA	1/0.9%	NA	1/0.1%	NA
FH	0/0.0%	NA	1/0.7%	2/0.2%	1/0.9%	NA	4/0.4%	NA
DE	0/0.0%	NA	1/0.7%	2/0.2%	1/0.9%	0/0.0%	1/0.1%	2/2.4%
DT	0/0.0%	1/0.3%	NA	NA	NA	NA	2/0.2%	NA

*R* success rate, *AE* adverse event, *TIA* transient ischemic attack, *PE* pericardial effusion, Tam tamponade, *MB* major bleeding, *FP* femoral pseudoaneurysm, *FH* femoral hematoma, *DE* device embolization, *DT* device thrombus

### DRT

**Fig. 4:**
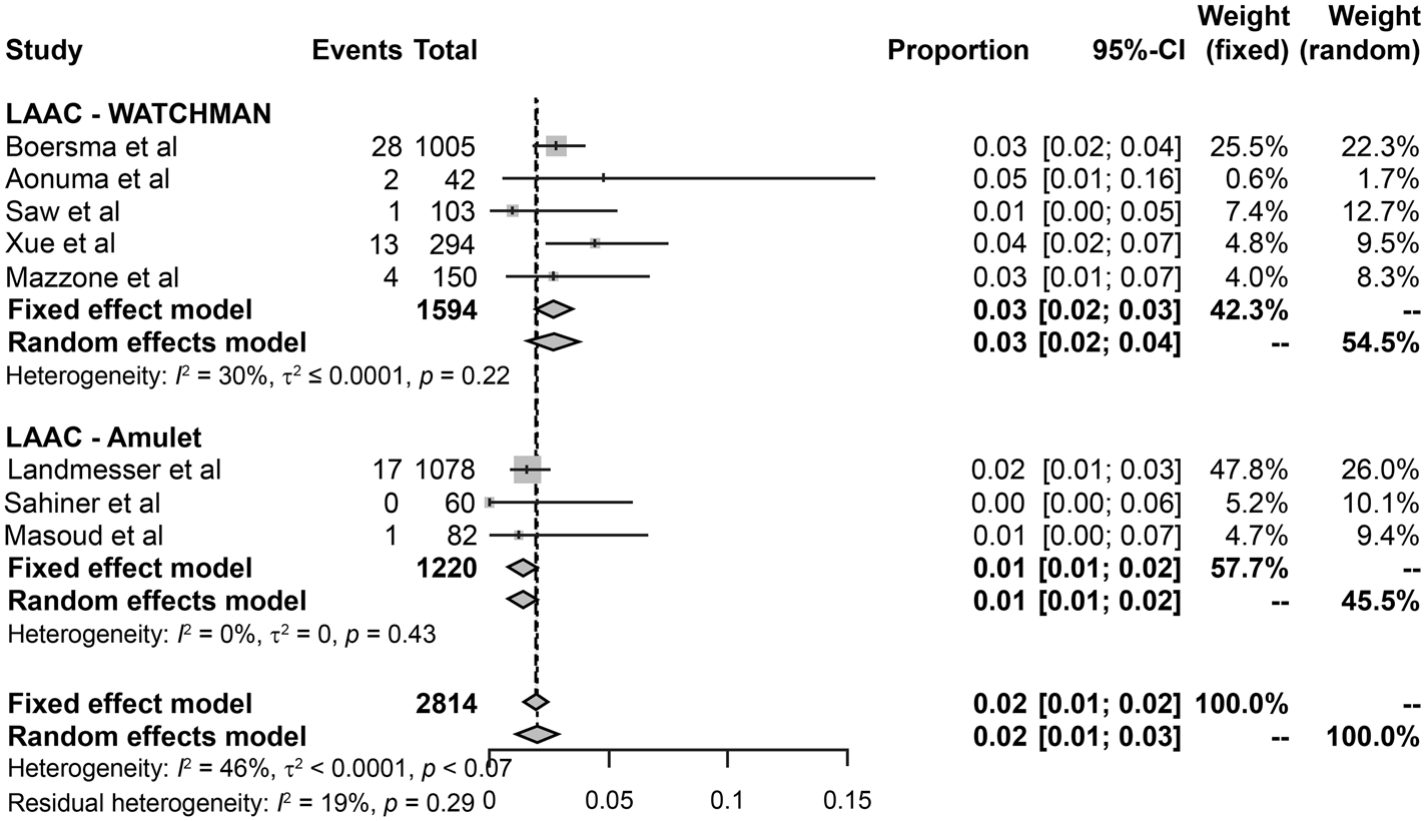
Forest plot of device-related thrombus rates in studies on WATCHMAN and Amulet. *CI* confidence interval

DRT was reported in eight LAAC device (WATCHMAN, 5; Amulet, 3) studies comprising 1482 and 1133 patients, respectively. The incidence rate of DRT was 2.3%, i.e., 66 cases in 2615 patients from eight LAAC device studies. The incidence of pooled DRT rates of WATCHMAN and Amulet were 0.03 (95% CI 0.02–0.04) and 0.01 (95% CI 0.01–0.02), respectively (Fig. 4). A lower heterogeneity between WATCHMAN and Amulet was observed (2814 person-years; *I*^2^ = 46). Most DRTs were detected on TEE either at follow-up or when symptoms occurred. A total of six complications out of 66 DRTs were observed in this study (rate of complication for DRT, 9%). In the WATCHMAN studies, two complications were found at 6 months in the study by Mazzone et al. [16], and one complication was found at 11 months in the study by Boersma et al. [17]. In the Amulet studies, three complications were reported by Landmesser et al. [20]; the first was found after a diagnosis of ischemic stroke conducted at 161 days, the second DRT was confirmed 243 days after the stroke, and the third was observed 24 days prior to the stroke, but no DRT was reported 37 days after the stroke occurred (Table 5). Regarding DRT, the incidence after WATCHMAN implantation was higher in the studies by Aonuma et al. [14] (4.8%) and Xue et al. [15] (4.4%) than in those by Mazzone et al. [16] (2.7%), Boersma et al. [17] (2.8%), and Saw et al. [18] (1%). Three studies on Amulet implantation found a lower incidence of DRT than that reported in studies on WATCHMAN, with Sahinar et al., Landmesser et al. and Masoud et al. reporting incidence of 0%, 1.6%, and 1.2%, respectively [19–21].

**Table 5:** Device-related thrombus events from selected LAAC studies

	WATCHMAN					Amulet		
Author	Aonuma et al. [14]	Xue et al. [15]	Mazzone et al. [16]	Boersma et al. [17]	Saw et al. [18]	Sahiner et al. [19]	Landmesser et al. [20]	Masoud et al. [21]
Patients	42	294	150	1005	103	59	1075	80
% FU	100%	100%	100%	89%	100%	100%	94%	81%
TEE FU	45 days and 6 months	45 days and 6 months	1, 6, 12 months	Variable	6 months	1, 6, 12 months	3, 12 months	2 months
DRT	2	13	4	28	1	0	17	1
%	4.8%	4.4%	2.7%	3.1%	1.0%	0.0%	1.6%	1.2%
Complications	0	NA	2	1	0	NA	3	NA
Condition	NA	Low compliance	NA	1 Stroke	NA	NA	1 at ischemic stroke 1 after stroke 1 prior to stroke	NA
When	2 at 6 months	4 at 45 days 9 at 180 days	2 at 6 months	28 at 11 months	1 at 6 months	NA	1 at 161 days 1 at 243 days 1 at 24 days*	1 at 2 months
Treated drug	2 Restart VKA	9 DAPT	Unknown	22 adjusted regimens, 6 no action	OAC	NA	10 DAPT, 1 ASA, 1 Clop, 2 OAC w/APT, 1 OAC, 2 LMWH	Apixaban
Result	NA	NA	NA	24 resolved included 5 no action	Resolved without sequela	NA	NA	Resolved

*FU* follow-up, *TEE* transesophageal echocardiography, *DRT* device-related thrombus, *ASA* aspirin, *Clop* clopidogrel, *VKA* warfarin, *DOAC* direct oral anticoagulant, *OAC* oral anticoagulant, *LMWH* low-molecular-weight heparin, *APT* antiplatelet therapy, *DAPT* dual antiplatelet therapy

*DRT observed 24 days prior to stroke and no DRT after stroke 37 days, and second DRT observed 113 days after stroke

### Follow-up and oral anticoagulant therapy after device implantation

**Fig. 5:**
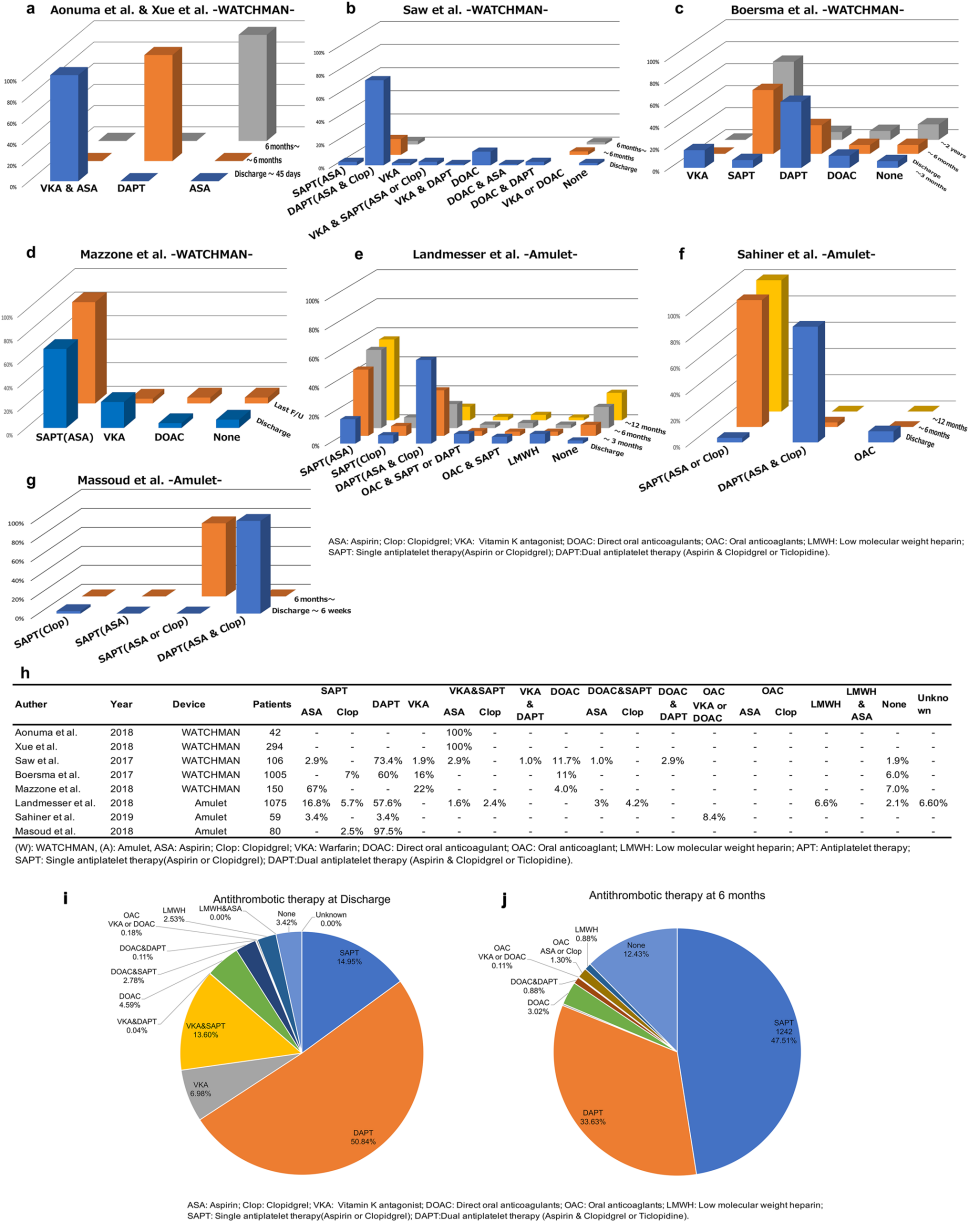
Medication after the implantation of left atrial appendage closure devices. **a** Aonuma et al. and Xue et al. (WATCHMAN), **b** Saw et al. (WATCHMAN), **c** Boersma et al. (WATCHMAN), **d** Mazzone et al. (WATCHMAN), **e** Landmesser et al. (Amulet), **f** Sahiner et al. (Amulet), **g** Masoud et al. (Amulet), **h**, **i** antithrombotic therapy on discharge, **j** antithrombotic therapy at 6 months

With respect to antithrombotic therapy after device implantation, this study found seven different dosing patterns in all eight studies; two WATCHMAN studies by Aonuma et al. and Xue et al. were strictly controlled by acetyl salicylic acid (ASA) and vitamin K antagonist (VKA) for 45 days after device implantation [14, 15]. Subsequently, the medication was changed to dual antiplatelet therapy (DAPT) at 6 months. In the other six studies, medication management was different (Fig. 5a–g). The most common strategy was DAPT (50.84%), followed by single antiplatelet therapy (SAPT) (14.95%) at discharge. Altogether, DAPT and SAPT accounted for 65.79% of the total 2808 patients. OAC (VKA or DOAC) (0.18%), DOAC and SAPT (2.78%), DOAC and DAPT (0.11%), and only DOAC (4.59%) were used. The total DOAC usage rate was 7.66%. This study found no increase in the use of DOAC in patients who had LAAC devices. Compared with medications at discharge, the medication management at the 6 months follow-up period was different. SAPT (47.51%) was the most common therapeutic strategy, followed by DAPT (33.63%). The usage rate of VKA or DOAC was 0.11%; DOAC alone, 3.02%; and DOAC and DAPT, 0.88%. No medication was administered to 325 patients (12.43%) of the total 2615 patients (Fig. 5h–j).

The follow-up period—including a general TEE—was 45-days, 3-months, or 6-months. For WATCHMAN, which was most commonly used, VKA and ASA were recommended until 45 days after implantation, and DAPT for up to 6 months after implantation, unless leaks were confirmed by TEE within 45 days. Implementation of the recommended administration method varied based on the time of discharge and the follow-up period. If no issues were detected on TEE, the patient was advised to take ASA for the rest of their life [18].

## Discussion

We analyzed the recent studies published in the last 5 years to assess the incidence of all-cause mortality, stroke, and major bleeding between LAAC devices and DOACs. Our analysis showed that the results were comparable among WATCHMAN, Amulet, and rivaroxaban. Post-implantation antithrombotic therapy protocols differed according to the devices used, with WATCHMAN using ASA and VKA for 45 days, before transition to DAPT. The Amulet used either SAPT or DAPT. Among the five WATCHMAN studies, the studies by Aonuma et al. [14] and Xue et al. [15] used two drugs, ASA and VKA, and the rates of DRT incidences were 4.8% and 4.4%, respectively. In the other three studies, SAPT or DAPT was used. Mazzone et al. [16] used SAPT and the rate of DRT incidences was 2.7%. Boersma et al. [17] and Saw et al. [18] mainly used DAPT and reported the rate of DRT were 3.1% and 1.0%, respectively. Among the three Amulet studies, the studies by Sahiner et al. [19], Landmesser et al. [20] and Massoud et al. [21] used SAPT or DAPT and the rates of DRT incidences were 0%, 1.6%, and 1.2% respectively (Table 5).

Previous studies of WATCHMAN have shown efficacy and safety in post-implantation anticoagulant therapy. In this study, the rate DRT incidences was lower in Amulet than in the WATCHMAN groups. However, in the study of Amulet by Peyrol et al., 27 patients (71.1%) and 10 patients (26.3%) were treated with DAPT and SAPT, respectively, instead of anticoagulant therapy. The overall incidence of DRT was 0 after administration and at follow-up [25]. The data implied that patients taking SAPT or DAPT with LAAC devices tended to show a relatively low incidence rate of DRT in comparison with VKA.

We found that DOAC usage was low for patients who had LAAC devices. Studies have shown that anticoagulant management strategy vary in patients with LAAC devices. There was a difference in the incidence of all-cause death, stroke, major bleeding, and DRT between devices, suggesting that their incidence is influenced by differing drug management between devices.

Tereshchenko et al., performed a network meta-analysis including 96,017 patients with nonvalvular AF from 21 RCTs with 29 study arms. They compared the efficacy and safety of DOACs (apixaban, dabigatran, edoxaban, rivaroxaban), VKA, ASA, and WATCHMAN to prevent thromboembolism in patients with nonvalvular AF. The authors concluded that the entire spectrum of therapies significantly reduced the incidence of stroke, systemic embolism events, and mortality in these patients. Rivaroxaban appeared to be most effective in preventing stroke and systemic embolism, and WATCHMAN was considered the most effective life-saving therapy [6]. This study did not include Amulet. In our study, Amulet showed a similar incidence of stroke and major bleeding to those with DOAC (Fig. 2b, c). The incidence of DRT tended to be higher in WATCHMAN studies wherein patients were treated with VKA and ASA after LAAC implantation, compared with its lower incidence in Amulet studies wherein patients were treated with DAPT or SAPT. We found that the usage rate of DOACs for patients with LAAC devices was lower than that of other drugs. This is different from the percentage of DOACs currently used as an alternative to VKA for stroke prevention in patients with AF. It was speculated that the benefits of LAAC devices may increase over time, as the implanted LAAC devices may eventually be covered with a neointima, which may reduce drug dosage. However, there is currently no clear evidence on anticoagulant therapy after implantation of LAAC devices. Therefore, it is currently administered at each facility according to the patient's condition. Patient management after LAAC implantation is important to achieve favorable clinical outcomes. This study found that there was room for optimizing medications specifically for each AF patient with LAAC. Patients with LAAC were given a variety of medications. The proportion of elderly people is increasing very rapidly in Japan; therefore, clinical studies focusing on medication dosage in LAAC patients are expected to be conducted to improve clinical outcomes and quality of life in patients with AF.

DRT has a certain probability of occurrence with any device. It is asymptomatic and is undetected unless it either leads to a stroke or the patient undergoes TEE during regular screening. The underlying cause of DRT is complicated, and various factors might be involved. Lempereur et al., conducted a systematic review of DRT in 2017 using the results of 30 studies from 2008 to 2015 with WATCHMAN, Amplatzer Cardiac Plug (ACP), and Amulet. The incidence of DRT was 3.8% (*n* = 2118) and 82 DRTs occurred. The median time of DRT diagnosis after implantation was 1.5 months, which occurred early. Most cases were diagnosed with TEE. However, the incidence of adverse events, such as neurological complications, was low, and the study concluded that anticoagulant treatment is safe and highly effective [26]. In addition, Garot et al. reported that while the rate of DRT incidences is generally 3–7%, there was a causal relationship between DRT and stroke or systemic embolism as stroke occurred within 2 months after DRT detection [27]. The incidence of DRT in our study was 2.3%, which was lower with Amulet than with WATCHMAN. Chun et al., conducted a single-center study in 2013, including 78 successfully-implanted WATCHMAN and ACP patients [28]. The patient characteristics in the two groups were as follows: CHA2DS2-VASc score, 4.1 ± 1.5 versus 4.5 ± 1.8; HAS-BLED score, 3.1 ± 1.1 versus 3.1 ± 1.1. Although the differences in these two scores were not significant, the incidence of DRT was clearly lower in the DAPT-treated group than in the VKA-treated group. Our study shows similar results when comparing patients in the WATCHMAN group who were treated with VKA after implantation with those in the Amulet group who were treated with DAPT; the incidence of DRT was 33% higher in the VKA-treated group. Furthermore, there are VKA and DAPT groups in the WATCHMAN group, but the incidence rate of DRT is clearly 75% higher in the VKA-treated group (Fig. 3). The frequent site where of thrombus development is between the left lateral ridge and the periphery of the screw that connects the cable attached to the delivery catheter of the device. The size of the thrombus is reported to be within 5–10 mm or < 5 mm. DRT may occur during the implantation procedure, although the incidence is reportedly low, and it disappears after 2 weeks of administration of low-molecular-weight heparin [28]. We could not find any association between the tendency for DRT occurrence and the management of antithrombotic therapy. Further device improvement, discontinuation of antithrombotic therapy, patient compliance issues, and frequency of assessment by regular TEE are important factors to consider when estimating the actual incidence of DRT [29, 30].

## Limitations

This study has several limitations. First, as this meta-analysis indirectly compared the incidence of all-cause mortality, stroke, and major bleeding between LAAC devices and DOACs, significant heterogeneity was present between the groups. Second, in the last 5 years, only studies using rivaroxaban as a DOAC were included. Third, the follow-up periods for detecting DRT occurrence using TEE were different between the studies. Fourth, in LAAC studies, data on gastrointestinal bleeding and intracranial bleeding were not consistently collected. Finally, various medications were administered to patients with LAAC devices as compared to patients with DOACs. Regarding the safety and efficacy of LAAC devices and DOACs, Osmancik et al. recently reported a multi-center randomized study in patients at high risk of stroke and bleeding. They found that LAAC devices were noninferior to DOACs in preventing major AF-related cardiovascular, neurological, and bleeding events. The patient population of LAAC devices and DOACs had similar backgrounds, with the CHA2DS2-VASc score of 4.7 and 4.7 and HAS-BLED score of 3.0 and 3.1 respectively. The types of AF were similar in the LAAC devices and DOACs (LAAC: 26.4% paroxysmal, 23.4% persistent, 9% long-standing persistent, and 41.3% permanent; DOAC: 33.3% paroxysmal, 22.9% persistent, 8% long-standing persistent, and 35.8% permanent) [31]. In our study, the study population of LAAC devices and DOACs had different backgrounds with the CHA2DS2-VASc score of 3.6 and 3.4, and HAS-BLED scores of 3.2 and 1.4 respectively. The type of AF in our study were different from Osmanicik’s study (WATCHMAN: 36% paroxysmal, 18% persistent, and 51% permanent; Amulet: 21% paroxysmal, 78% persistent, and 67% permanent; DOAC: 39% paroxysmal, 24% persistent, and 25% permanent) (Table 3). Given these differences in AF characteristics and risk scores between LAAC devices and DOACs, the results of our study do not definitively confirm the safety and efficacy of LAAC devices and DOACs. Nevertheless, this study is the first systematic review and meta-analysis that shows the rates of all-cause mortality, stroke, and major bleeding with WATCHMAN, Amulet, and rivaroxaban based on recent studies from the last 5 years, and shows the incidences of DRT, complications, and current medication managements in AF patients with LAAC devices.

## Conclusions

This meta-analysis of most recent studies in the past 5 years indicated that the incidence of all-cause mortality, stroke, and major bleeding were similar between the two catheterized minimally invasive LAAC devices and DOACs among patients with AF. The rates of complications were acceptable, and the rates of DRT incidences were lower than the average reported by other previous studies. However, further follow-up is needed. This study suggests that the strategy of timing and dosing anticoagulant and antiplatelet therapies should be further evaluated to find an optimal medical regimen for patients with AF with LAAC devices. LAAC devices may become an alternative treatment that can allow for the reduction of antithrombotic therapy use in patients with AF who are at a high risk of bleeding, are ineligible for long-term therapy, or have contraindications to DOACs.

## Data Availability

All data generated or analyzed during this study are included in this published article.
